# Emerging roles of selenium on metabolism and type 2 diabetes

**DOI:** 10.3389/fnut.2022.1027629

**Published:** 2022-11-10

**Authors:** Jiuxiang Zhao, Hong Zou, Yanling Huo, Xiaoyi Wei, Yu Li

**Affiliations:** CAS Engineering Laboratory for Nutrition, Shanghai Institute of Nutrition and Health, University of Chinese Academy of Sciences, Chinese Academy of Sciences, Shanghai, China

**Keywords:** selenium, dietary intake, glucose and lipid metabolism, type 2 diabetes, selenoproteins

## Abstract

Selenium is recognized as an essential element for human health and enters human body mainly *via* diet. Selenium is a key constituent in selenoproteins, which exert essential biological functions, including antioxidant and anti-inflammatory effects. Several selenoproteins including glutathione peroxidases, selenoprotein P and selenoprotein S are known to play roles in the regulation of type 2 diabetes. Although there is a close association between certain selenoproteins with glucose metabolism or insulin resistance, the relationship between selenium and type 2 diabetes is complex and remains uncertain. Here we review recent advances in the field with an emphasis on roles of selenium on metabolism and type 2 diabetes. Understanding the association between selenium and type 2 diabetes is important for developing clinical practice guidelines, establishing and implementing effective public health policies, and ultimately combating relative health issues.

## Introduction

Selenium is a natural chemical element existing in soil, water, and air. Selenium reaches the human body by food chain through incorporation into plants, animals, and aquatic organisms (1). From the public health perspective, selenium is treated as an essential micronutrient and is commonly used in dietary supplementation products widely consumed in western countries (2, 3). In human beings, the nutritional functions of selenium are achieved by 25 selenoproteins (4), with essential enzymatic functions, including hydroperoxide/phospholipid peroxide reduction (glutathione peroxidases, GPxs) (5, 6), thiol redox status regulation (thioredoxin reductases) (7), thyroid hormone activity regulation (iodothyronine deiodinases, Dios) (8), selenium transport (selenoprotein P, SelP) (9) and some are yet to be determined. Selenium is reported to play a role in maintaining redox balance, anti-cancer, and improving immunity, and is closely related to Keshan disease, diabetes, mental disorder, inflammation, and infections (10, 11).

Diabetes is a highly costly chronic disease (12), with an estimated worldwide prevalence of 463 million adults according to the 2019 report of the International Diabetes Federation Diabetes Atlas (IDF) (13). Type 2 diabetes accounts for 90% of all diabetes and is characterized by defective insulin secretion and/or insulin resistance (14). Although the mechanisms of insulin resistance and type 2 diabetes remain not fully understood, accumulating evidence suggests that oxidative stress plays an important role in both onset and progress (15). As several selenoproteins have the potential to protect the body from oxidative stress, selenium is expected to be protective against type 2 diabetes (10, 16). However, recent evidence raised concerns that a high level of selenium exposure may be associated with an increased risk of type 2 diabetes (17–20).

Even though how selenium affects the risk of type 2 diabetes is conflicting, evidence has confirmed that people with low status may benefit from additional selenium intake (21, 22). Although, selenium is assumed to be helpful in the prevention and therapy of type 2 diabetes (23, 24), selenium intake, including selenium supplementation, should be excluded for primary or secondary diabetes prevention in populations with adequate selenium status (25). This review focuses on the association of dietary selenium with type 2 diabetes epidemiology and discusses the major selenoproteins in the regulation of glucose and lipid metabolism and their implication in the development of type 2 diabetes.

## Selenium in food

**FIGURE 1:**
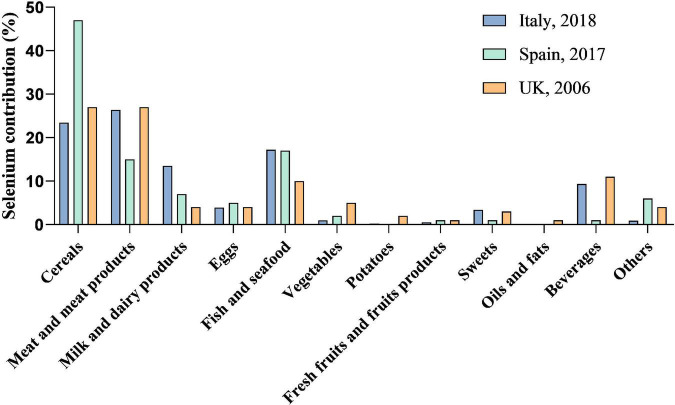
Selenium contribution to total dietary selenium intake. Data for selenium contribution to total dietary selenium intake are adapted from published studies. Diet is the main source of selenium for human beings. The species of food, selenium content in food, as well as the amount taken in diet affect the daily selenium intake and selenium status.

Diet is a major source of selenium for humans and the content of selenium in foods varies greatly (Figure 1). The main source of selenium in diet are cereals because of the large amounts consumed as well as meats and seafood with high protein contents (26). Also, plant-based sources of selenium, including wheat, pearl millet, and maize, are more effective in reversing the deleterious effects of selenium deficiency (27). Natural fruits generally contain low amounts of selenium, rarely exceeding 10 μg/kg and vegetables with a maximum concentration of 6 μg/kg (28). From the selected investigation, the main food sources in diet of selenium intake include cereals, meat products, milk and dairy products, beverages, fish and seafood (29–31). All these groups provide more than 85% of the selenium intake. In addition, ready-to-eat meals, vegetables, fruits, sweets, and beverages contribute to a small part of the dietary selenium intake. Processing technology could affect selenium content in food and bioaccessibility of selenium species, among which soaking, fermentation were reported to increase the bioaccessible selenium content and heating declined the bioaccessibility of SeMet and SeCys (32, 33). Another selenium source of exposure is well-known as selenium supplementation. Considering the low abundance of selenium in daily foods, consuming a diet with natural selenium concentrations is not abundant. Hence, scientific works have dealt with selenium-biofortification strategies to obtain selenium-enriched food or feed, *via* plant cultivation (soil fertilization with inorganic selenium), animal feeding (with selenium-enriched plants), microorganism transformation (fermentation) (34, 35). Asides from the classical methods, pharmacological products and nano-selenium applications can also increase the selenium concentration in the human body (36, 37).

Not only the total intake of dietary selenium but also the selenium species ingested is important to human health (38). Selenium exists in inorganic and organic forms and intakes of different foods are correlated with different types of selenium species. Organic selenium forms in foods mainly contain selenomethionine (SeMet), selenocysteine (SeCys), selenium-methylselenocysteine (SeMeCys), and gamma-glutamyl-Se-methylselenocysteine (GGMSC), and drinking water mainly contains inorganic selenium species such as selenate and selenite (36, 39). Efficient uptake and metabolism of dietary selenium primarily depend on its chemical forms (40). Organic selenium is more easily absorbed by the human body compared with inorganic selenium (41), and more than 90% of SeMet is absorbed in human body but only about 50% of selenium is from selenite. In addition, nano-selenium attracts growing attention due to its high chemical stability, biocompatibility, and low toxicity (42, 43). Nano-selenium has been applied as antioxidants, dietary supplements, antidiabetic agents (37, 44).

Selenium has long been termed “an essential poison” as selenium doses exceeding 400 μg/day may exert toxic actions according to the World Health Organization (WHO) (45). The recommended daily allowance (RDA) of selenium varies hugely depending on the geographical area, ranging from 25 μg/day for adult women in Japan up to 100 μg/day in the Netherlands and Macedonia, but most RDA levels are in the range of 50–60 μg/day. Both selenium deficiency and excess have been associated with adverse health effects, and the health effects of selenium are recognized as the inextricable U-shaped link with status (10). The selenium content in diabetes serum is commonly lower than normal ones, while additional concerns were raised about the diabetes risk associated with selenium intake above the RDA (55 μg/day) (46). Overall, selenium exposure adds to type 2 diabetes risk across a wide range, especially above dietary intake of 80 μg/day and blood selenium of 120 μg/L (17). Whereas people with low selenium status may benefit from additional selenium intake, those with adequate-to-high selenium status might be affected adversely and should not take selenium supplements.

## Selenium in metabolism

### Selenoproteins

The biological actions of selenium are mainly mediated by selenoproteins. Selenium is integrated into selenoproteins in the form of selenocysteine. To date, 25 selenoproteins have been identified in humans, among which glutathione peroxidase 1 (GPx1), selenoprotein P (SelP), selenoprotein S (SelS), selenoprotein V (SelV), and iodothyronine deiodinases (Dios) have been reported to associate with glucose and lipid homeostasis (Table 1) (47, 48).

**TABLE 1:** Role of main selenoproteins associated with metabolism and type 2 diabetes.

Selenoproteins	Tissue distribution	Cellular location	Physiological function	Health effects on diabetes
Cytosolic glutathione peroxidase 1(GPx1)	Ubiquitous, highly expressed in erythrocytes, liver, kidney, lung	Cytosol and mitochondria	GPx1 reduces intracellular hydrogen peroxide and lipid peroxides	Overquenching intracellular reactive oxygen species, regulating the concentration of hydrogen peroxide (49)
Selenoprotein P (SelP)	Expressed in the liver, heart, brain, and kidney	Secreted into the plasma	SelP functions as a selenium-transporter and maintains selenium homeostasis and possesses antioxidant activity	Promoting insulin resistance (50)
Selenoprotein S (SelS)	Plasma, various tissues	Endoplasmic reticulum (ER) membrane and plasma membrane	SelS promotes ER-associated degradation of errant proteins to increase the translocation of misfolded proteins to the cytosol	Antioxidant protection and anti-ER stress effects in the pancreas (51), up-regulating glucose utilization and down-regulating glucose output in the liver (52) Positively correlates with serum amyloid A in skeletal muscle (53) Positively correlates with HOMA-IR in adipose tissue (54) Negatively correlates with fasting plasma glucose in serum (55)
Selenoprotein V (SelV)	Testis (mainly in seminiferous tubules)	Cytoplasm and nuclei	Regulation of body selenium metabolism and lipid metabolism	Inhibitor of body fat accumulation and activator of energy expenditure (56) Protection against endoplasmic reticulum stress and oxidative injury induced by pro-oxidants (57)
Iodothyronine deiodinases (Dios)	Dio1: liver, kidney, and thyroid Dio3: placenta, brain, gastrointestinal tract, skin, and liver Dio2: pituitary, brain, brown adipose tissue, skeletal muscle, thyroid, heart, and ear	Dio2: endoplasmic reticulum membrane Dio1 and Dio3: the plasma membrane	Thyroid hormone-regulating iodothyronine deodinase	Regulation of energy homeostasis (58)

GPx1 is localized in the cytosol and mitochondria. This enzyme can catalyze the reduction of hydrogen peroxide (H_2_O_2_) and lipid hydroperoxides using GSH as a reducing cofactor (59). GPx1 is highly sensitive to changes in both selenium status and oxidative stress conditions (60). GPx1 is involved in regulating insulin synthesis and secretion, insulin sensitivity, glucose and lipid homeostasis and the onset and progression of diabetes (61). SelP functions as a selenium-transport protein to deliver selenium from the liver to other tissues to maintain appropriate selenium levels in tissues. Selenium is necessary for the synthesis of antioxidative selenoproteins. Thus, SelP plays an important role in the cellular antioxidative system by maintaining these selenoproteins. Furthermore, SelP possesses multifunctional properties such as GPx-like antioxidant enzyme activity (62), peroxynitrite scavenging (63), and metal-binding activity (64). SelS is resident in the endoplasmic reticulum (ER). It is involved in regulating oxidative stress, ER stress, and inflammatory response (51, 52, 65). SelS plays roles in antioxidant protection and anti-ER stress in the pancreas and blood vessels, while it promotes insulin resistance in the liver, adipose tissue, and skeletal muscle (51, 66). SelV is highly expressed in the testis (mainly in seminiferous tubules). It is involved in regulating body selenium metabolism and lipid metabolism (56, 57). Dios, including Dio1, Dio2, and Dio3, are thyroid hormone-regulating iodothyronine deiodinase. Dios are expressed in multiple tissues. Dio1 and Dio3 are located in the plasma membrane and Dio2 is located in the endoplasmic reticulum membrane. Dios play an important role in thyroid hormone signaling involving many key reactions in energy homeostasis and individual growth and development (58, 67).

### Glucose metabolism

Selenoproteins are involved in regulating glucose metabolism. Early studies found that inorganic selenium showed an insulin-like effect. Sodium selenate promoted glucose transport and glucose metabolism through the mitogen-activated protein/myelin basic protein kinases (MAPK) and ribosomal S6 protein kinases in rat adipocytes at very high doses (68–70).

Most selenoproteins are antioxidant enzymes and play roles in maintaining insulin secretion with their antioxidant activity. SelP is relatively highly expressed in pancreatic islets, which acts as an antioxidant to protect β cells (42). GPx1 can degrade intracellular H_2_O_2_ (59). In pancreatic islets, GPx1 reduces the damage of H_2_O_2_ on β cells and promotes the normal secretion of insulin.

SelP synthesis is regulated like a gluconeogenic enzyme in the liver. SelP gene expression is regulated by the interaction of the transcription factors FoxO1 and HNF-4α with the co-activator PGC-1α (71, 72). These transcription factors similarly control the expression of gluconeogenic enzymes: G6PC (glucose-6-phosphatase, G6Pase, catalytic subunit) and PCK1 (phosphoenolpyruvate carboxykinase, PEPCK), which are involved in hepatic glucose release to adapt to feeding and fasting (73, 74). It was found that liver SelP mRNA levels have been shown to increase during fasting and decrease after feeding in mice, which indicated that the liver could fine-tune SelP secretion according to the nutritional state (72). Furthermore, SelP transcription can be inhibited by insulin *via* the PI3K/Akt/FoxO1 pathway (72, 75). Insulin-induced phosphorylation of FoxO1 results in its nuclear exclusion and inhibition of FoxO1-dependent transcription of SelP in liver cells (71). Thus, insulin-mediated regulation of hepatic SelP production and secretion represents a physiological link between selenium homeostasis and carbohydrate metabolism (71).

Selenium deficient diet results in impaired islet function, low insulin secretion, and high blood glucose (76, 77). The deficiency of selenium decreases the expression of several selenoprotein genes and proteins in different tissues, which may dysregulate glucose homeostasis. It was reported that GPx1 deficiency induced type 1 diabetes-like phenotype (61). GPx1-knockout-mice developed islets β cell damage and insulin reduction (78, 79). Hepatic-specific deletion of SelS in mice caused obesity, hepatic steatosis, insulin resistance, and disturbed glucose homeostasis (66, 80). It was reported that reduced synthesis of selenoproteins, including GPx1 and MsrB1, caused by overexpression of an i(6)A(-) mutant selenocysteine tRNA promoted glucose intolerance and led to a diabetes-like phenotype (81). Selenocysteine lyase (Scly) is the enzyme that supplies selenium for selenoprotein biosynthesis *via* decomposition of the amino acid selenocysteine (82). Moreover, it was found that Scly knockout mice fed with low selenium dietary reduced GPx1 and SelS protein levels and affected hepatic glucose homeostasis (83). Taken together, selenium and selenoproteins play important roles in glucose metabolism, especially in maintaining a redox balance to promote the normal synthesis and secretion of insulin.

### Lipid metabolism

Selenoproteins are also involved in regulating hepatic lipid accumulation. It was reported that SelS expression was down-regulated in the liver in high-fat diet (HFD)-fed mice and db/db mice, and SelS expression levels were reduced in the PA-induced primary hepatocytes (66).

Hepatic triglyceride synthesis consists of fatty acid uptake and *de novo* lipogenesis (84, 85). It was reported that serum free fatty acids level was elevated in hepatocyte-specific SelS knockout (SelSH-KO) mice, and the expression levels of cluster of differentiation 36 (CD36), fatty acid transport protein 2 (FATP2) and fatty acid transport protein 5 (FATP5), which are involved in fatty acid uptake, were markedly increased in the liver of SelSH-KO mice (66). On the other hand, the expression levels of peroxisome proliferator-activated receptor α (PPARα), carnitine palmitoyltransferase 2 (CPT2), and acyl-coenzyme A oxidase 1 (ACOX1), which are responsible for fatty acid oxidation, were down-regulated in SelSH-KO mice. These results suggested that hepatic SelS deletion increased hepatic triglyceride and diacylglycerol accumulation *via* promoting fatty acid uptake and reducing fatty acid oxidation (66).

FGF21 is an endocrine hepatokine produced predominantly in the liver (86, 87). Hepatic-specific deletion of SelS (SelS LKO) decreased the production of hepatokine FGF21 and adipokine adiponectin and increased adipose tissue size. These results indicated that the FGF21-adiponectin axis was inhibited in SelS LKO mice, which exacerbated hepatic metabolic disorders (66). Taken together, these studies show that selenoproteins participate in regulating lipid metabolism, especially in lipid intake and fatty acid oxidation, which indicates that selenoproteins may be a potential intervention target for lipid metabolic disorders.

## Epidemiology of selenium and type 2 diabetes

**FIGURE 2:**
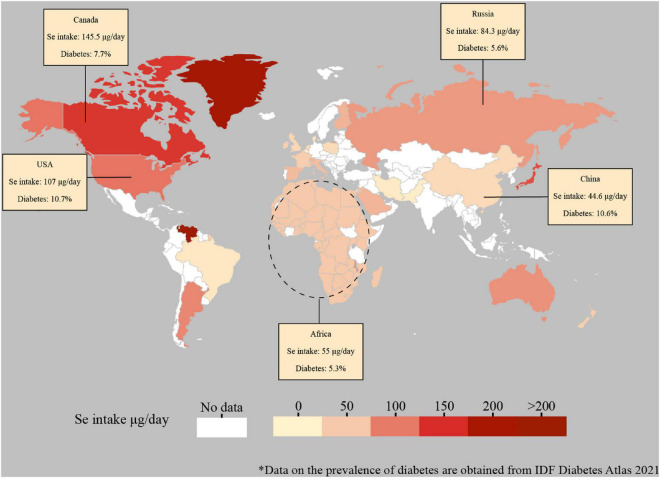
Global estimated daily intake of selenium and prevalence of diabetes. Data for the global estimated daily selenium intake are adapted from the published studies (95-102). The selenium data of mainland China was selected. The selenium data of Africa was adapted from the Africa selenium daily supplied amount. Data for the prevalence of diabetes (data included until 2021) are derived from the Diabetes Atlas of the International Diabetes Federation (https://diabetesatlas.org/data/en/indicators/2/). Dashed lines are not supposed to accurately represent regions.

Selenium is expected to protect against type 2 diabetes because of the potential of several selenoproteins to protect against oxidative stress (19, 88, 89). Selenium intake varies greatly among countries due to the selenium differences in local soil and foods consumed (Figure 2). The relationship between selenium level and the prevalence of type 2 diabetes is possibly U-shaped, with possible adverse effects occurring both below and above the physiological range for optimal activity of some or all selenoproteins (90). Whereas dietary selenium supplement has been applied to improve glucose metabolism, accumulating evidence showed that exposure to a high level of selenium increased the risk of type 2 diabetes. In a multivariate logistic regression model, an increase of 10 μg/L in selenium induced to the prevalence of diabetes mellitus by 12% (91), showing a dose-dependent relationship between selenium level and diabetes. Higher serum selenium was discovered to be linked with increased plasma glucose levels and glycosylated hemoglobin levels (92). Intake of high-level selenium might affect the expression and(or) function of key regulators for glycolysis, gluconeogenesis, and lipogenesis (20). Furthermore, the association between selenium and type 2 diabetes was independent of insulin resistance at high serum selenium levels (19, 91). The prevalence of diabetes, as well as glucose and glycosylated hemoglobin levels, increased with increasing selenium concentrations up to 140 μg/L of selenium exposure (93), while several studies insisted on a risk serum selenium level of 160 μg/L (17, 94). In addition, the non-experimental studies reached agreement with the findings from randomized controlled trials, illustrated that selenium exposure from moderate to high levels is associated with increased risk for type 2 diabetes. Further research is required to clarify the optimal range of selenium intake and status for minimizing the potential adverse effects on glucose metabolism and preventing type 2 diabetes.

## Mechanisms of selenium in type 2 diabetes

**FIGURE 3:**
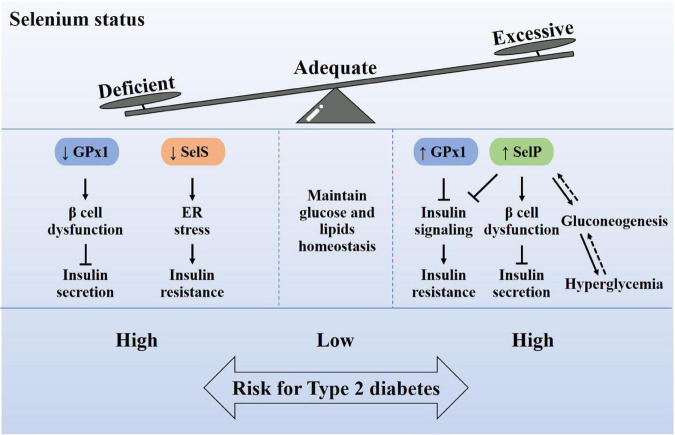
Relationship between selenium homeostasis and type 2 diabetes. Adequate selenium intake is very important for maintaining the homeostasis of glucose and lipid metabolism. Excessive or insufficient selenium intake will cause the increase or decrease of selenoproteins in the body, which in turn leads to a high risk of type 2 diabetes. The abnormal content of selenoproteins, including Gpx1, SelP, and SelS, may cause oxidative damage of β cells, insulin signaling impairment, endoplasmic reticulum stress and gluconeogenesis, which induce insulin secretion defects or insulin resistance. Therefore, it is recommended to supplement selenium according to the state of selenium.

Type 2 diabetes is characterized by defective insulin secretion and/or insulin resistance, and the potential molecular mechanisms include interference of oxidative stress, insulin signaling, gluconeogenesis, and endoplasmic reticulum (ER) stress (Figure 3). Abnormal selenium status affects the occurrence and development of diabetes through these mechanisms.

### β cells and pancreas

Pancreas β cells have a fragile antioxidant system. The expression levels of antioxidant enzymes in pancreatic islets are substantially lower compared with various other tissues, which renders β cells sensitive toward oxidative and nitrosative stress (103). Severe antioxidant selenoproteins deficiency may result in oxidative damage of β cells and lower insulin secretion. GPx1-knockout-mice fed a high-fat diet for 12 weeks decreased plasma insulin and glucose-induced insulin secretion (78, 79). It was reported that GPx1-knockout-mice elevated islet superoxide and hydroperoxide production and up-regulated p53 phosphorylation. By contrast, after overexpressing GPx1 in pancreatic β cells, C57BLKS/J mice were protected from the β cell damage when stimulated with streptozotocin, and db/db mice exhibited reversed hypoinsulinemia and hyperglycemia (104). However, global overexpression of GPx1 induced obesity, hyperglycemia, insulin resistance in mice, and developed type 2 diabetes-like phenotypes (105–107), which infered adverse effects of excessive selenoprotein biosynthesis and the complexity of redox status. The results of SelP further prove this point. It has been shown that excess SelP impairs the function of pancreatic β cells and decreases insulin secretion (50, 108). The injection of purified human SelP protein in mice resulted in a decrease in insulin levels, a decline of β cells and α-cells in the pancreas, and also a rearrangement of the position of these cells in the pancreatic islets (50). Furthermore, the administration of SelP-neutralizing antibodies could improve insulin secretion and glucose intolerance in a mouse model of diabetes (50). Thus, selenium homeostasis and redox balance are extremely important for β cells and insulin secretion.

### Insulin signaling

The antioxidant activity of selenoproteins can protect the islets from oxidative stress, but excessive antioxidant activity is not beneficial for insulin signaling. The binding of insulin to its receptor activates NADPH oxidase enzymes and results in the production of space H_2_O_2_ (109). These small amounts space of H_2_O_2_ can act as second messengers and are required to deactivate two insulin-signaling inhibitors: tyrosine phosphatase 1B (PTP-1B) and phosphatase and tensin homolog protein (PTEN). This H_2_O_2_-mediated deactivation is considered to enhance the insulin-induced PI3K/Akt signaling, which facilitates glucose uptake, inhibits glycogen synthesis, and suppresses gluconeogenesis (110). GPx1 can degrade intracellular H_2_O_2_ and regulate its concentration (59). However, when intracellular physiological H_2_O_2_ is eliminated by excessive activity of GPx1, insulin signaling may be impaired. In this regard, GPx1-knockout-mice were protected from insulin resistance induced by a high-fat diet due to increased H_2_O_2_ production and inactivation of PTEN (111). Conversely, mice over-expressing GPx1 exhibited insulin resistance and hyperinsulinemia (105–107). Therefore, the appropriate amount of selenium and selenoproteins will benefit insulin function. Based on the control of SelP transcription through PGC-1α/FoxO1/HNF-4α, it was found insulin could inhibit SelP transcription by the PI3K/Akt/FoxO1 pathway (75). High levels of SelP impaired insulin signaling and dysregulated glucose metabolism both in the liver and muscle *via* the inactivation of adenosine monophosphate-activated protein kinase (AMPK) (72, 112). So, SelP has been identified as a “hepatokine” that induces insulin resistance and excess SelP promotes type 2 diabetes (50, 108). Thus, a high level of selenoproteins may impair insulin sensitivity through the interference of the insulin signaling cascade.

### Gluconeogenesis

Plasma SelP levels were reported elevated in patients with type 2 diabetes, and there was an association between high plasma selenium and fasting plasma glucose in type 2 diabetes patients (47, 113, 114). SelP and gluconeogenic enzyme gene expression are similarly regulated by methylation of the same transcription factors (71, 72). SelP, together with G6PC and PCK1 is transcribed through PGC-1α/FoxO1/HNF-4α. Under the normal metabolic condition, insulin inactivates the transcription of SelP and gluconeogenic enzymes. Under the condition of high glucose and insulin resistance, the dysregulated transcriptional activity of FoxO1 enhances the biosynthesis of SelP and gluconeogenic enzymes, which results in elevated plasma SelP and selenium levels and further elevated plasma glucose levels (72, 115, 116). Thus, from this point, elevated plasma SelP levels might be considered as the result rather than the cause of hyperglycemia and insulin resistance (117).

### Endoplasmic reticulum stress

Chronic endoplasmic reticulum (ER) stress affects glucolipid metabolism, which is crucial to the occurrence and development of insulin resistance and nonalcoholic fatty liver disease (NAFLD) (118, 119). ER stress is induced by unfolded or misfolded proteins accumulated in the ER, which initiates unfolded protein response (UPR) to restore homeostasis in the ER. ER-associated protein degradation (ERAD) is activated to remove unfolded or misfolded proteins (120). Selenoproteins, such as SelS and SelK, are induced under ER stress, which play important roles in ERAD and ER stress. Mechanistically, SelS forms a multiprotein complex with degradation in endoplasmic reticulum protein 1 (Derlin1)-ubiquitin ligase E3-p97ATPase and SelK to participate in ERAD, which mediates misfolded proteins in the ER to be translocated back into the cytosol for degradation by the proteasome (121–123). Consistently, knockdown of SelS increased the expression of ER stress marker genes (124, 125), whereas its overexpression protected against ER stress injury in hepatocytes and cell lines (65, 66, 125, 126). Consistently, ER stress was increased in SelS-hepatic-knockout mice and SelS-knockdown hepatocytes, but suppressed in SelS-overexpress hepatocytes (66). It was likely that excessive misfolded or unfolded proteins were accumulated in SelS deficiency hepatocytes due to impaired ERAD capability, resulting in chronic ER stress (66). Collectively, these results show evidence supporting that SelS has the potential to reduce ER stress injury and may protect hepatocytes from the development of insulin resistance and hepatic steatosis.

## Conclusion and future perspectives

In conclusion, we systemically review the role of selenium and selenoproteins in type 2 diabetes and indicate the therapeutic potential of selenium supplementation in the treatment of metabolic disorders. Even though the interaction of some other selenoproteins with type 2 diabetes has not been verified, their effective roles in the regulation of glucose and lipid metabolism are becoming increasingly clear. Although there have been some inconsistent results, extensive evidence has suggested that selenium supplementation is beneficial for preventing and treating several chronic diseases (127). Future studies are needed to explore the association between selenium exposure and metabolic effects in more details with selenium exposure, and the potential mechanisms.

Selenium supply is very important for maintaining glucose and lipid homeostasis in healthy adults and patients with type 2 diabetes. However, the epidemiology of observational and experimental studies of selenium in type 2 diabetes reveal that both selenium deficiency and severe excess lead to insulin resistance and β cell dysfunction, with potential molecular mechanisms including interference of oxidative stress, insulin signaling, gluconeogenesis, and ER stress. Thus, selenium should be supplemented according to the status of selenium, while excessive selenium supplement is not recommended.

Nutrigenetic research has identified several single nucleotide polymorphisms in selenoproteins, which may clarify the high variability of selenium nutritional status in different populations (34, 43) and influence metabolic parameters in response to selenium supplementation (44). Thus, more personalized nutritional recommendations are needed to consider not only the regional particularities but also the genetic characteristics of the population or individuals.

## Author contributions

JZ, HZ, and YL researched data for the manuscript, made substantial contributions to discussions of the content, and wrote the manuscript. YH and XW edited the manuscript with important intellectual content. All authors reviewed and edited the manuscript before submission.
